# Utérus de Couvelaire: aspect impressionnant mais utérus fonctionnel

**DOI:** 10.11604/pamj.2016.25.11.10606

**Published:** 2016-09-19

**Authors:** Mehdi Kehila, Rim Ben Hmid

**Affiliations:** 1 Service C du Centre de Maternité et de Néonatologie de Tunis, Faculté de Médecine de Tunis, Université Tunis El Manar, Tunisie

**Keywords:** Hématome rétroplacentaire, apoplexie utérine, Couvelaire, Retroplacental haematoma, uterine apoplexy, Couvelaire

## Image en médecine

Il s’agit d’une patiente âgée de 32 ans, primigeste, qui a consulté à 28 semaines d’aménorrhée dans un tableau typique d’hématome rétro-placentaire compliqué d’une mort fœtale in utéro. Une échographie obstétricale faite au bloc opératoire a confirmé la mort fœtale in utéro. Les biométries étaient en rapport avec le terme et il n’y avait pas de chevauchement des os du crane; ce qui était en faveur d’une mort récente. Il y avait une image échogène rétro placentaire arrondie faisant 8 cm de diamètre évoquant devant ce tableau un hématome rétro-placentaire. La NFS a montré une hémoglobine à 9 g/dl. Le bilan d’hémostase était normal. La patiente était instable de point de vu hémodynamique; une évacuation par voie haute a été décidée. Une césarienne réalisée sous anesthésie générale, par voie de Pfannenstiel, a révélé un utérus sous tension, oedématié, bleuté dans son ensemble (Utérus de couvelaire). L’hystérotomie, a mis en évidence du sang rouge vif, un placenta décollé et un hématome rétroplacentaire de 600 g. Le fœtus était en état de mort apparente et a pesé 700 g. Après l’évacuation de la grossesse, l’utérus avait un aspect bleuté quasiment dans sa totalité mais se rétractait de façon correcte. Après hystérorraphie Il persistait un saignement modéré rouge vif qui a tari suite à la mise en route de perfusion de prostaglandines (Nalador^®^). Les suites opératoires étaient simples.

**Figure 1:**
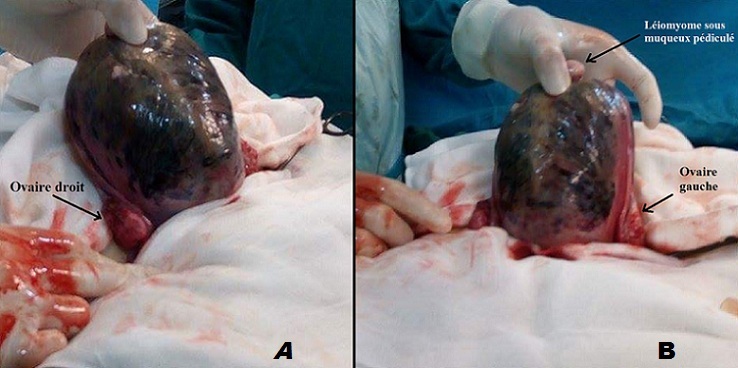
(A et B) aspect bleuté de l’utérus, secondaire à des suffusions hémorragiques dans l’épaisseur du myomètre donnant l’aspect de l’utérus de Couvelaire

